# Detecting Cooperativity between Transcription Factors Based on Functional Coherence and Similarity of Their Target Gene Sets

**DOI:** 10.1371/journal.pone.0162931

**Published:** 2016-09-13

**Authors:** Wei-Sheng Wu, Fu-Jou Lai

**Affiliations:** Department of Electrical Engineering, National Cheng Kung University, Tainan, Taiwan; Università degli Studi di Milano, ITALY

## Abstract

In eukaryotic cells, transcriptional regulation of gene expression is usually achieved by cooperative transcription factors (TFs). Therefore, knowing cooperative TFs is the first step toward uncovering the molecular mechanisms of gene expression regulation. Many algorithms based on different rationales have been proposed to predict cooperative TF pairs in yeast. Although various types of rationales have been used in the existing algorithms, functional coherence is not yet used. This prompts us to develop a new algorithm based on functional coherence and similarity of the target gene sets to identify cooperative TF pairs in yeast. The proposed algorithm predicted 40 cooperative TF pairs. Among them, three (Pdc2-Thi2, Hot1-Msn1 and Leu3-Met28) are novel predictions, which have not been predicted by any existing algorithms. Strikingly, two (Pdc2-Thi2 and Hot1-Msn1) of the three novel predictions have been experimentally validated, demonstrating the power of the proposed algorithm. Moreover, we show that the predictions of the proposed algorithm are more biologically meaningful than the predictions of 17 existing algorithms under four evaluation indices. In summary, our study suggests that new algorithms based on novel rationales are worthy of developing for detecting previously unidentifiable cooperative TF pairs.

## Introduction

Transcription factors (TFs) are a kind of proteins whose biological functions are to transcriptionally regulate the expression of their target genes. In eukaryotic cells, transcriptional regulation of gene expression is usually not achieved by a TF alone but by cooperative TFs which function together to precisely control the location, time and amount of gene expression [1–3]. Therefore, knowing cooperative TFs is crucial for studying the molecular mechanisms of transcriptional regulation of gene expression.

Many algorithms have been proposed to identify cooperative TF pairs in yeast [4–20]. Different algorithms are developed based on different rationales and their performances vary under different evaluation criteria [21–24]. For example, two algorithms [4,6] assume that the genes bound by both TFs of a cooperative TF pair are more co-expressed or closer in the protein-protein interaction network than are genes bound by either TF alone. Another five algorithms [5,11,14,18,20] assume that for a cooperative TF pair, their binding sites have shorter distance, are more co-depleted of nucleosomes or co-occur more often than expected by chance. Some other algorithms [15,16,18,19] assume that the observed number of the shared target genes of a cooperative TF pair is higher than random expectation (see Table 1 for details). Apart from the above mentioned algorithms which aim to identify cooperative TF pairs in yeast, several advanced algorithms have been proposed to identify cooperative TF pairs in human [25–27].

**Table 1:** The rationales of 17 existing algorithms.

Authors	The rationale of the existing algorithm for predicting cooperative TF pairs (CTFPs)	# of predicted CTFPs
Banerjee and Zhang [4]	For a CTFP, the genes bound by both TFs should be more co-expressed than are the genes bound by either TF alone.	31
Harbison et al. [5]	For a CTFP, their binding sites should co-occur more often within the same promoters than would be expected by chance.	94
Nagamine et al. [6]	For a CTFP, the genes bound by both TFs should be closer in the protein-protein interaction network than are the genes bound by either TF alone.	24
Tsai et al. [7]	For a CTFP, their interaction effect (estimated using ANOVA) should significantly influence the expression of genes bound by both TFs.	18
Chang et al. [8]	A stochastic system model is developed to assess TF cooperativity.	55
He et al. [9]	The multivariate statistical method, ANOVA, is used to test whether the expressions of the target genes were significantly influenced by the cooperative effect of their TFs.	30
Wang [10]	Pairwise mixed graphical models or Gaussian graphical models are used for identifying combinatorial regulation of TFs.	14
Yu et al. [11]	An algorithm called Motif-PIE is developed for predicting interacting TF pairs based on the co-occurrence of their binding motifs and the distance between the motifs in promoter sequences.	300
Elati et al. [12]	A data mining technique called LICORN is developed for deriving cooperative regulations.	20
Datta and Zhao [13]	Log-linear models are used to study cooperative bindings among TFs.	25
Chuang et al. [14]	For a CTFP, the distance between their binding sites (in the promoter of their common target genes) should be significantly closer than expected by chance.	13
Wang et al. [15]	A Bayesian network framework is presented to reconstruct a high-confidence whole-genome map of transcriptional cooperativity in Saccharomyces cerevisiae by integrating a comprehensive list of 15 genomic features.	159
Yang et al. [16]	CTFPs are predicted by identifying the most statistically significant overlap of target genes regulated by two TFs in ChIP-chip data and TF knockout data.	186
Chen et al. [17]	A method called simTFBS is developed for inferring TF-TF interactions by incorporating motif discovery as a fundamental step when detecting overlapping targets of TFs based on ChIP-chip data.	221
Lai et al. [18]	For a CTFP, (i) the two TFs should have a significantly higher number of common target genes than random expectation and (ii) their binding sites (in the promoters of their common target genes) should tend to be co-depleted of nucleosomes in order to make these binding sites simultaneously accessible to TF binding.	27
Wu and Lai [19]	For a CTFP, the overlap of the targets (defined by TF binding and TF perturbation data) of these two TFs should be higher than random expectation.	50
Spivak and Stormo [20]	For a CTFP, the distribution of nucleotide spacings between their binding sites should be deviated significantly from random expectation.	1399

Although various types of rationales have been used in the existing algorithms, the functional coherence is not yet used. This prompts us to develop a new algorithm based on the functional coherence and similarity of the target gene sets. First, the proposed algorithm assumes that the common target genes of two cooperative TFs have similar functions. This rationale is biologically plausible since co-regulated genes are known to have similar functions [28–30]. Second, the proposed algorithm assumes that two cooperative TFs have similar target gene sets. Since the biological role of two cooperative TFs is to co-regulate the expression of a set of genes, they should have a significant number of shared target genes [5,11,15,16,18,19]. In other words, the target gene sets of two cooperative TFs should be similar to each other.

## Materials and Methods

### Data sources

Two data sources were used in this study. First, the experimentally validated target genes of 151 TFs were retrieved from the YEASTRACT database [31]. The association between a TF and its target gene was supported by two types of experimental evidence. One is the TF binding (TFB) evidence from the detailed gene by gene band-shift, foot-printing experiments or the high throughput genome-wide ChIP-chip experiments showing that the TF binds to the promoter of its target gene. The other one is the TF regulation (TFR) evidence from the detailed gene by gene analysis or the genome-wide expression analysis showing that the perturbation (knockout or over-expression) of the TF-encoding gene causes a significant change in the expression of its target gene. Therefore, the target genes of a TF retrieved from the YEASTRACT database are of biological significance since they are validated by two types of experimental evidence.

The second data source used in this study is the functional similarity scores of all gene pairs in yeast retrieved from Yang et al.’s study [32]. Yang et al. proposed an improving Gene Ontology (GO) semantic similarity measure based on downward random walks to calculate the functional similarity score of any gene pair. Their score has been shown to be more biologically meaningful than the other existing functional similarity scores [32].

### The proposed algorithm

The proposed algorithm for identifying cooperative TF pairs is based on two rationales (functional coherence and similarity of the target gene sets). First, the proposed algorithm assumes that the common target genes of two cooperative TFs have similar functions. This rationale is biologically plausible since co-regulated genes are known to have similar functions [28–30]. Second, the proposed algorithm assumes that two cooperative TFs have similar target gene sets. Since the biological role of two cooperative TFs is to co-regulate the expression of a set of genes, they should have a significant number of shared target genes [5,11,15,16,18,19]. In other words, the target gene sets of two cooperative TFs should be similar to each other.

**Fig 1:**
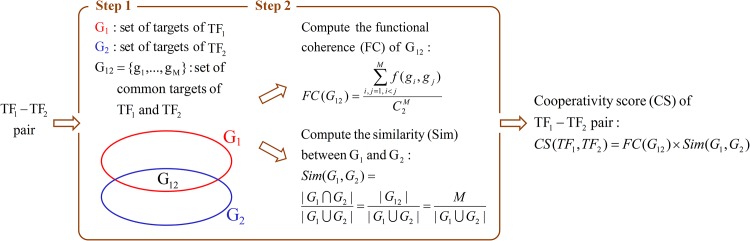
The proposed two-step procedure of calculating the cooperativity score of a TF pair (TF_1_-TF_2_).

Fig 1 depicts the proposed two-step procedure of calculating the cooperativity score of a TF pair (e.g. TF_1_-TF_2_). The first step is to retrieve the set of TF_1_’s target genes (denoted as G_1_), the set of TF_2_’s target genes (denoted as G_2_) and the set of the common target genes of TF_1_ and TF_2_ (denoted as G_12_) from YEASTRACT database [31]. Note that G_1_, G_2_ and G_12_ are of biological significance since the regulatory associations between a TF and its target genes are validated by two types of experimental evidence (TFB evidence and TFR evidence). The second step is to calculate the cooperative score of TF_1_-TF_2_ based on the functional coherence of G_12_ and the similarity between G_1_ and G_2_.

The functional coherence (FC) of G_12_ is calculated using the following formula

$$FC\left(G_{12}\right)=\frac{\sum_{i,j=1,i<j}^{M}f\left(g_{i},g_{j}\right)}{C_{2}^{M}}$$

where *M* is the number of genes in *G*_12_ = {*g*_1_, ⋯, *g*_*M*_}, $C_{2}^{M}$ is all possible gene pairs formed by genes in G_12_, and *f*(*g*_*i*_, *g*_*j*_) is the functional similarity score of *g*_*i*_ and *g*_*j*_ retrieved from Yang et al.’s study [32]. Note that *FC*(*G*_12_) is actually the average of the functional similarity scores of all possible gene pairs formed by genes in G_12_. The higher the *FC*(*G*_12_) is, the higher the functional coherence of the genes in G_12_ is.

The similarity (Sim) between G_1_ and G_2_ is calculated using Jaccard similarity coefficient

$$Sim\left(G_{1},G_{2}\right)=\frac{\left|G_{1}\cap G_{2}\right|}{\left|G_{1}\cup G_{2}\right|}=\frac{\left|G_{12}\right|}{\left|G_{1}\cup G_{2}\right|}=\frac{M}{\left|G_{1}\cup G_{2}\right|}$$

where *M* = |*G*_12_| is the number of genes in G_12_ and |*G*_1_ ∪ *G*_2_| is the number of genes in the union of G_1_ and G_2_. The higher the *Sim*(*G*_1_, *G*_2_) is, the higher the similarity between G_1_ and G_2_ is. Then the cooperativity score (CS) of TF_1_-TF_2_ is calculated using the following formula

$$CS\left(TF_{1},TF_{2}\right)=FC\left(G_{12}\right)\times Sim\left(G_{1},G_{2}\right)=\frac{\sum_{i,j=1,i<j}^{M}f\left(g_{i},g_{j}\right)}{C_{2}^{M}}\times \frac{M}{\left|G_{1}\cup G_{2}\right|}$$

The higher the *CS*(*TF*_1_, *TF*_2_) is, the higher the cooperativity between TF_1_ and TF_2_ is.

Since we can retrieve the experimentally validated target genes of 151 TFs from YEASTRACT database [31], the cooperativity scores of 11325 (151*150/2) TF pairs can be calculated. Finally, these 11325 TF pairs are sorted by their cooperativity scores, where the top one TF pair has the highest cooperativity score and therefore is the most plausible cooperative TF pair. That is, the finally output of the proposed algorithm is a ranked list of 11325 TF pairs, where the top one TF pair is the most plausible cooperative TF pair.

### Four existing evaluation indices

To judge the biological significance of the set of predicted cooperative TF pairs (PCTFPs) from an algorithm, here we adopt the following four existing evaluation indices.

#### Index 1: The statistical significance of the overlap with the benchmark set

Yang et al. [16] proposed to evaluate the performance of an algorithm by calculating the significance of the overlap of its set of PCTFPs with a benchmark set of 27 known cooperative TF pairs collected from MIPS transcription complex catalog [33]. The significance of the overlap is represented as −logP, where P is the p-value computed using Fisher exact test [34]. The higher the −logP is, the better the performance of an algorithm is.

#### Index 2: The co-regulatory coefficient of a PCTFP

Balaji et al. [35] proposed the co-regulatory coefficient to evaluate the biological plausibility of a PCTFP. The co-regulatory coefficient represents the significance of a PCTFP in regulating common target genes. The greater the co-regulatory coefficient is, the higher the biological plausibility of a PCTFP is. To evaluate the biological significance of the set of PCTFPs from an algorithm, we used the average of the co-regulatory coefficients of all PCTFPs from an algorithm. The higher the average is, the better the performance of an algorithm is.

#### Index 3: The shortest path length of a PCTFP in the physical protein-protein interaction network

Aguilar and Oliva [36] observed that a cooperative TF pair has a shorter path length in the physical protein-protein interaction (PPI) network (using PPI data from BioGRID database [37]) than expected by random. Therefore, the greater the reciprocal of the shortest path length of a PCTFP in the PPI network is, the higher the biological plausibility of a PCTFP is. To evaluate the biological significance of the set of PCTFPs from an algorithm, we used the average of the reciprocals of the shortest path lengths of all PCTFPs from an algorithm. The higher the average is, the better the performance of an algorithm is.

#### Index 4: The functional similarity of a PCTFP

Lai et al. [21] proposed to evaluate the biological plausibility of a PCTFP by using the functional similarity between the two TFs of a PCTFP. The functional similarity scores between any two TFs were retrieved from Yang et al.’s study [32]. The higher the functional similarity score between the two TFs of a PCTFP is, the higher the biological plausibility of a PCTFP is. To evaluate the biological significance of the set of PCTFPs from an algorithm, we used the average of the functional similarity scores of all PCTFPs from an algorithm. The higher the average is, the better the performance of an algorithm is.

## Results and Discussion

From “The proposed algorithm” subsection, it is known that the final output of the proposed algorithm is a ranked list of 11325 TF pairs, where the top one TF pair is the most plausible cooperative TF pair. Here we consider the top 40 TF pairs as the PCTFPs from the proposed algorithm. Considering the top 40 TF pairs is reasonable because the number of the PCTFPs from most (>10) existing algorithms [4,6–10,12–14,18,19] falls between 13 and 60 (see Table 1).

### Validation of the 40 PCTFPs from the proposed algorithm

To judge the biological plausibility of each of the 40 PCTFPs from the proposed algorithm, we provide five types of validation (see Table 2 for details). The five types of validation are (i) whether a PCTFP is predicted by any existing algorithm, (ii) whether a PCTFP has physical or genetic interaction, (iii) whether both TFs of a PCTFP are studied in the same experimental publications, (iv) whether a PCTFP has common GO terms, and (v) whether a PCTFP has common target genes.

**Table 2:** Five types of validation of the 40 PCTFPs from the proposed algorithm.

PCTFP		Evidence of the cooperativity between TF1 and TF2				
TF1	TF2	Algorithm Evidence	Physical/Genetic Evidence	Co-citations	# of Common GO Terms	# of Common Targets
Arg80	Arg81	7	5	47	5	8
Ifh1	Sfp1	1	0	16	4	82
Met28	Met31	2	1	29	8	11
Hap2	Hap4	5	3	100	8	18
Met32	Met4	5	6	43	6	30
Met31	Met32	6	8	54	14	18
Hap3	Hap5	5	5	65	10	4
Met31	Met4	5	5	42	6	14
Met28	Met4	3	7	35	10	13
**Pdc2**	**Thi2**	0	0	6	5	2
Met28	Met32	3	0	33	9	14
Mig1	Mig2	3	7	67	14	4
Ifh1	Rap1	1	1	22	4	105
Gcr1	Gcr2	3	10	26	7	8
Hap3	Hap4	1	3	93	8	7
Fhl1	Ifh1	1	7	33	7	26
Rap1	Sfp1	7	0	36	6	113
Hap2	Hap3	3	4	118	9	7
Hap4	Hap5	3	2	59	8	6
Aft1	Aft2	5	9	63	6	15
Stp1	Stp2	2	5	40	9	2
Mbp1	Swi6	13	12	147	7	14
**Hot1**	**Msn1**	0	0	13	3	2
Gal4	Gal80	3	34	185	6	2
Gcr2	Tye7	1	5	7	3	6
Pdr1	Pdr3	5	12	187	10	30
Dal81	Stp2	1	1	17	3	2
Ino2	Ino4	6	13	117	10	10
Cbf1	Met4	5	5	39	7	24
Ace2	Swi5	12	3	99	9	30
Oaf1	Pip2	4	6	57	13	13
Cbf1	Met32	5	1	38	8	23
Dal80	Dal81	1	0	28	7	4
Dal81	Gln3	2	0	23	7	9
Msn2	Sok2	2	2	36	6	150
Ste12	Tec1	6	12	114	9	171
Msn2	Yap1	3	2	114	7	143
**Leu3**	**Met28**	0	0	13	6	3
Swi4	Swi6	14	29	256	7	21
Bas1	Pho2	3	7	52	6	7

A PCTFP in boldface means that it is a novel CTFP predicted by the proposed algorithm. “Algorithm Evidence” provides the number of existing algorithms which predict the PCTFP. “Physical/Genetic Evidence” provides the number the experimental papers which suggest that the two TFs of the PCTFP have physical or genetic interaction. “Co-citations” provides the number of experimental papers which study the biological roles of both TFs of the PCTFP. More details could be seen at http://cosbi2.ee.ncku.edu.tw/40TFI/.

Overall speaking, the 40 PCTFPs from the proposed algorithm are likely to be biologically meaningful since (i) 93% (37/40) PCTFPs are also predicted by at least one existing algorithm, (ii) 80% (32/40) PCTFPs have physical or genetic interactions, (iii) the two TFs of each of the 40 PCTFPs are studied in the same experimental publications, (iv) 100% (40/40) PCTFPs have common GO terms, and (v) 100% (40/40) PCTFPs have common target genes.

Among the 40 PCTFPs from the proposed algorithm, three (Pdc2-Thi2, Hot1-Msn1 and Leu3-Met28) are novel predictions, which have not been predicted by any existing algorithms. Strikingly, Thi2 is known to act together with Pdc2 to respond to thiaminediphosphate demand [38]. Moreover, it is known that osmotic stress-induced gene expression requires both Hot1 and Msn1 [39]. The fact that two (Pdc2-Thi2 and Hot1-Msn1) of the three novel predictions have been experimentally validated in the literature [38,39] demonstrates the power of the proposed algorithm.

### Performance comparison of the proposed algorithm with 17 existing algorithms

**Fig 2:**
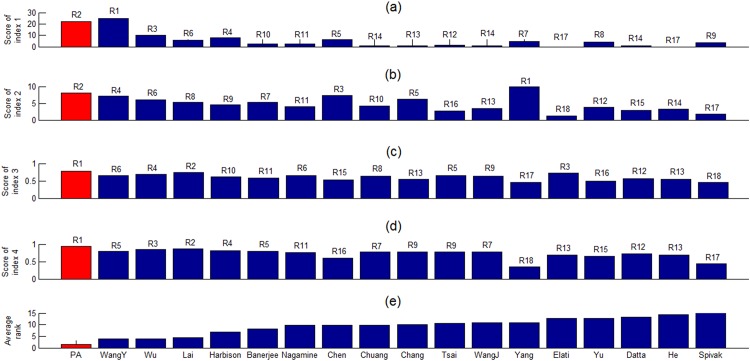
The performance comparison of the proposed algorithm and 17 existing algorithms in the literature. Performance comparison of the proposed algorithm and 17 existing algorithms using four existing evaluation indices. The performance comparison results using (a) index 1, (b) index 2, (c) index 3 and (d) index 4 are shown, where Rj means that the algorithm is ranked j among the 18 compared algorithms. For example, the proposed algorithm ranks first (R1) using the evaluation index 4 since the proposed algorithm has the largest score calculated using index 4. (e) The average rank is used to give the overall performance of an algorithm under four different evaluation indices. The average rank of an algorithm is the average of the ranks of an algorithm under four evaluation indices. For example, the average rank of the proposed algorithm is 1.5 = (2+2+1+1)/4 and the average rank of WangY’s algorithm is 4 = (1+4+6+5)/4. The smaller the average rank is, the better the performance of an algorithm is. Since the proposed algorithm has the smallest average rank, the overall performance of the proposed algorithm is the best among all the 18 compared algorithms.

Using four existing evaluation indices [16,21,35,36], we evaluate the biological significance of the PCTFPs from the proposed algorithms and those from the 17 existing algorithms. The PCTFPs of the 17 existing algorithms were retrieved directly from the corresponding papers [4–20]. Fig 2 shows that the proposed algorithm has the smallest average rank among the 17 compared algorithms, suggesting that the proposed algorithm is the best performing algorithm. That is, the PCTFPs from the proposed algorithms are more biologically meaningful than are the PCTFPs from the 17 existing algorithms.

### Robustness against the number of chosen PCTFPs

**Fig 3:**
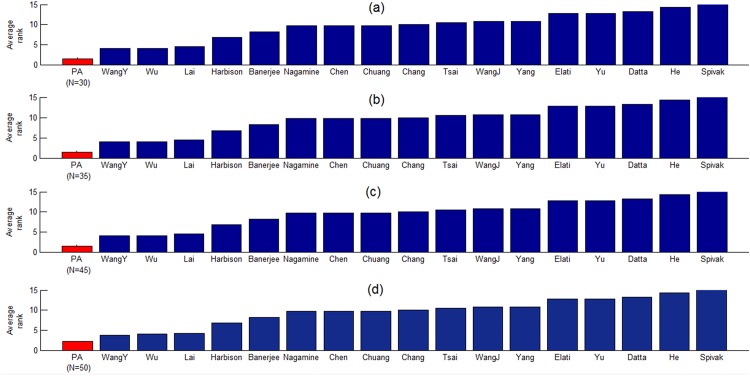
Robustness analysis of the proposed algorithm. The average rank of the proposed algorithm using top N, where (a) N = 30, (b) N = 35, (c) N = 45, and (d) N = 50, TF pairs of the ranked list of 11325 TF pairs as the PCTFPs from the proposed algorithm. It can be seen that no matter which value of N is used, the proposed algorithm always has the smallest average rank. That is, the PCTFPs from the proposed algorithm are always more biologically meaningful than those from the 17 existing algorithms. This suggests that the proposed algorithm is robust against the number of chosen PCTFPs.

In the last subsection, the 40 PCTFPs (i.e. the top 40 TF pairs of the ranked list of 11325 TF pairs) from the proposed algorithm are shown to be more biologically meaningful than those from the 17 existing algorithms in the literature. To check the robustness of the proposed algorithm against the number of chosen PCTFPs, we evaluate the performance of the proposed algorithm when choosing top N (N = 30, 35, 45 or 50) TF pairs as the PCTFPs from the proposed algorithm. Fig 3 shows that no matter which value of N is used, the proposed algorithm always has a smaller average rank than do the 17 existing algorithms in the literature. This suggests that the proposed algorithm is indeed robust against the number of chosen PCTFPs.

**Fig 4:**
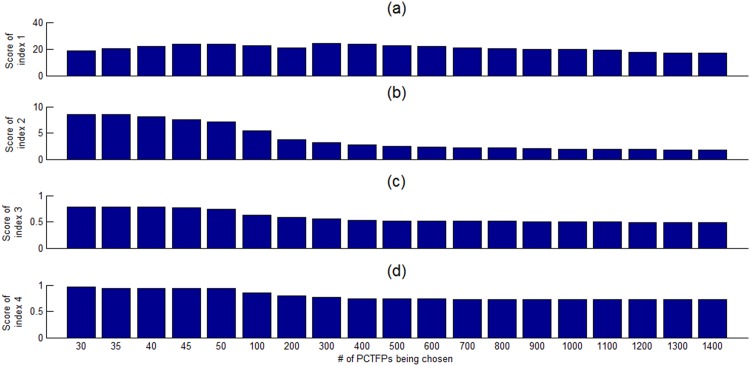
The scores of the four evaluation measures (shown in (a), (b), (c), (d)) with different top N (N = 30, 35, 40, …) chosen for the proposed algorithm.

Note that our algorithm and most existing algorithms identified less than 100 PCTFPs, but Spivak and Stormo’s algorithm [20] identified 1399 PCTFPs (see Table 1). It can be seen in Fig 2, Spivak and Stormo’s algorithm performs worst among all the compared algorithms. A possible reason is that their 1399 PCTFPs probably include a large number of false positives. It would be interesting to investigate how the performance of our algorithm evolves with larger N values. As shown in Fig 4, the scores of the four evaluation measures gradually decrease with larger N values, indicating a performance degradation of our algorithm with larger N values. Just like many false positives inside the 1399 PCTFPs from Spivak and Stormo’s algorithm, our PCTFPs probably include a large number of false positives with larger N values.

## Conclusions

In this study, we develop a new algorithm based on functional coherence and similarity of the target gene sets to identify cooperative TF pairs in yeast. The proposed algorithm provides 40 predicted cooperative TF pairs (PCTFPs) and the biological significance of the PCTFPs is validated by five types of validation. Among the 40 PCTFPs, three (Pdc2-Thi2, Hot1-Msn1 and Leu3-Met28) are novel predictions, which have not been predicted by any existing algorithms. Strikingly, two (Pdc2-Thi2 and Hot1-Msn1) of the three novel predictions have been experimentally validated in the literature, demonstrating the power of the proposed algorithm. Moreover, we show that the predictions of the proposed algorithm are more biologically meaningful than the predictions of 17 existing algorithms under four evaluation indices. In summary, our study suggests that new algorithms based on novel rationales (e.g. functional coherence) are worthy of developing for detecting previously unidentifiable cooperative TF pairs.
